# Structural Insights into Plant Viruses Revealed by Small-Angle X-ray Scattering and Atomic Force Microscopy

**DOI:** 10.3390/v16030427

**Published:** 2024-03-10

**Authors:** Eleonora V. Shtykova, Evgeniy V. Dubrovin, Alexander L. Ksenofontov, Polina K. Gifer, Maxim V. Petoukhov, Valeriy K. Tokhtar, Irina M. Sapozhnikova, Andrey N. Stavrianidi, Larisa V. Kordyukova, Oleg V. Batishchev

**Affiliations:** 1 National Research Centre, “Kurchatov Institute”, Moscow 123098, Russia; shtykova@ns.crys.ras.ru (E.V.S.);; 2 Frumkin Institute of Physical Chemistry and Electrochemistry, Russian Academy of Sciences, Moscow 119071, Russia; dubrovin@polly.phys.msu.ru (E.V.D.); gifer.pk@phystech.edu (P.K.G.); stavrianidi.andrey@gmail.com (A.N.S.); 3 Faculty of Physics, Lomonosov Moscow State University, Moscow 119991, Russia; 4 Belozersky Institute of Physico-Chemical Biology, Lomonosov Moscow State University, Moscow 119991, Russia; ksenofon@belozersky.msu.ru; 5 Scientific and Educational Center, Botanical Garden of the National Research University “BelSU”, Belgorod 308033, Russia; tokhtar@bsu.edu.ru; 6 Institute of Chemical Engineering, Ural Federal University Named after the First President of Russia B. N. Yeltsin, Ekaterinburg 620002, Russia; i.m.sapozhnikova@urfu.ru

**Keywords:** helical plant viruses, rod-shaped viruses, flexible filamentous viruses, viral structure, viral proteins, nanoparticles, bionanotechnology, vaccine development, small-angle X-ray scattering, atomic force microscopy

## Abstract

The structural study of plant viruses is of great importance to reduce the damage caused by these agricultural pathogens and to support their biotechnological applications. Nowadays, X-ray crystallography, NMR spectroscopy and cryo-electron microscopy are well accepted methods to obtain the 3D protein structure with the best resolution. However, for large and complex supramolecular structures such as plant viruses, especially flexible filamentous ones, there are a number of technical limitations to resolving their native structure in solution. In addition, they do not allow us to obtain structural information about dynamics and interactions with physiological partners. For these purposes, small-angle X-ray scattering (SAXS) and atomic force microscopy (AFM) are well established. In this review, we have outlined the main principles of these two methods and demonstrated their advantages for structural studies of plant viruses of different shapes with relatively high spatial resolution. In addition, we have demonstrated the ability of AFM to obtain information on the mechanical properties of the virus particles that are inaccessible to other experimental techniques. We believe that these under-appreciated approaches, especially when used in combination, are valuable tools for studying a wide variety of helical plant viruses, many of which cannot be resolved by classical structural methods.

## 1. Introduction

A large number of plant viruses are currently known, many of which are dangerous pathogens. This explains the great interest in their structural and functional research aimed at creating virus-resistant agricultural crops, which is particularly important in developing countries [1,2]. Plant viruses can be distinguished by many parameters, including the size of their virions, the nature of their genomes (RNA- or DNA-containing, single- or double-stranded), the symmetry of their capsids, the hosts they infect, the principles of their reproduction, etc. In terms of morphology, the virions may be rigid rod-shaped or flexible filamentous, spherical or bacilliform, with helical or icosahedral symmetry, and the viral particles of some virus species may even contain lipid membranes, which is more common for animal viruses.

The greatest diversity of species is found among non-enveloped plant viruses with helical symmetry of their capsids. Helical plant viruses are potentially useful for bionanotechnology to produce heterologous proteins in plants [3,4], to express epitopes of various human and animal pathogens for vaccine production or drug delivery [5,6]. Nowadays, some helical plant viruses are considered as promising platforms for the development of various nanomaterials for modern medicine, microelectronics, and other fields of human activities [7,8].

Helical plant viruses can be divided into two major groups: rod-like viruses and flexuous/flexible filamentous viruses [9]. Rod-shaped viruses belong to the family Virgaviridae, while filamentous viruses belong to the families Alphaflexiviridae, Potyviridae, Betaflexiviridae, and Closteroviridae. Filamentous viruses are significantly more abundant in nature than rod-shaped viruses [1], which may reflect their higher evolutionary fitness in the flowering plants that currently dominate the biosphere [9,10].

The flexible filamentous viruses have positive-sense, single-stranded RNA (ssRNA(+)) genomes which are encapsidated in non-enveloped particles [11,12] approximately 10–15 nm in diameter and several hundred nm long, depending on the length of the genome. Their particles are formed by the oligomerization of viral coat protein (CP) subunits arranged in a helical fashion to protect the viral genome.

The best-known representative of rigid rod-shaped viruses is tobacco mosaic virus (TMV), which belongs to the family Virgaviridae. Among the best known flexuous filamentous viruses are potato virus X (PVX) [13] and potato virus Y (PVY) [14], which belong to potex- or poty-virus families, respectively. The lengths of their virions are 515 and 730 nm, respectively, with a diameter of about 13 nm. Their coat proteins contain 237 and 267 amino acid residues, respectively. Importantly, according to a number of physicochemical methods and molecular modeling, the CPs of filamentous viruses are partially disordered, which greatly complicates their structural study [15,16].

The structure of rod-like viruses has been well characterized in 1980s by X-ray crystallography using the TMV model [10,17,18]. This method has retained its primacy in resolution for many decades, and has been modified as the complexity of the species has increased [19,20]. In contrast to rigid, rod-shaped plant viruses such as TMV, X-ray crystallography is not suitable for resolving the 3D structure of filamentous plant viruses, e.g., potex or potyviruses. Instead, the most informative structural studies have come from X-ray fiber diffraction and electron microscopy (EM) [10,21]. In the early 21st century, transmission EM (TEM) has been used to determine the shape and size of various virions [21]. The next step is the use of negative staining to construct the 3D images of virions and, in particular, the viral RNA.

There is still limited direct data on the architecture of whole flexible virions or whole CPs of these viruses at high resolution. Structure determination of flexible viruses in general is a major challenge for both NMR and X-ray crystallography. The cryo-EM approach, which also allows to obtain high-resolution 3D structures, works better with icosahedral [22,23,24] or rigid rod-shaped helical plant viruses [25], while the virions of flexible viruses have mostly been studied by low-resolution structural methods, such as various optical methods, tritium planigraphy or negative staining TEM [9,10,11].

In general, the structure of helical plant viruses is thought to be much more labile than previously thought [10]. This lability is associated with structural transitions, remodeling and the existence of alternative structural forms of the virions. It facilitates cell entry, viral assembly and disassembly, cell exit, and viral transport within the plant [11]. This lability, which gives viruses a number of advantages when infecting the host plant, makes them difficult to study. The difficulties in using high-resolution approaches to resolve the spatial structure of flexible filamentous viruses are mainly due to the flexibility of their virions and the high proportion of intrinsically disordered regions within the CP molecules.

The first high-resolution crystal structure of the CP fragment of the potexvirus papaya mosaic virus (PapMV) has only been obtained in 2012 [26]. Later, fragments of the CPs of the potexviruses bamboo mosaic virus (BaMV) [27], pepino mosaic virus (PepMV) [28] and potato virus X (PVX) [13], as well as the potyviruses watermelon mosaic virus (WMV) [29], turnip mosaic virus (TuMV) [30], and potato virus Y (PVY) [14], have been determined by cryo-EM. These CP structures revealed the existence of a conserved three-domain architecture common to all studied potexviruses and potyviruses, and the central parts of the CPs appeared to be structurally homologous. However, the structures of the flexible disordered N-/C-terminal domains have not been resolved. The IHRSR algorithm developed by Egelman [31] has been used for further helical reconstructions. Recently, cryo-EM and stability analyses have been carried out on virus-like particles (VLP) from representatives of the genera *Potyvirus* and *Ipomovirus* of the family Potyviridae, again lacking the N-terminal 91 or 66 amino acid residues, respectively, and a sequence-based prediction of the N-terminal domain has been made using AlphaFold to obtain the full-length structure [32].

So, for all their advantages, most of the “classic” structural techniques mentioned above have a number of disadvantages. Firstly, these methods are a kind of “averaging”, which may not be representative of larger objects. Secondly, the conditions under which the experiments are carried out are still not physiological. Thirdly, there is no way to follow the dynamics of processes, such as the stages of assembly or disassembly of the virus. To overcome these shortcomings, alternative physicochemical approaches have begun to be applied, which, although not of the same “high resolution” as, e.g., X-ray crystallography and cryo-EM, are more universal/suitable for structural studies of different shapes and complexes formed by biological objects in solution. Such approaches, which have provided essential information on the 3D structure and self-association of coat proteins within filamentous virus particles, include small-angle X-ray scattering (SAXS) or small-angle neutron scattering (SANS) analysis [33,34,35].

These methods are based on the effects of the elastic interaction of X-rays with bound electrons in samples in the case of SAXS and the interaction of neutrons with nuclei in the case of SANS.

Small-angle scattering is generally a universal diffraction technique used to study the supramolecular structure of matter with a resolution of about 1–2 nm, with respect to radiation sources and wavelength λ. The experimental small-angle scattering curve reflects the inverse relationship between the size of the scattering object and the scattering angle: the larger the size of the scattering object, the smaller the angles at which it scatters the incident beam. This relationship makes it possible to identify inhomogeneities of various sizes in the scattering material and thereby determine its shape and internal structure.

Due to the random or partially ordered orientation of dissolved molecules, spatial averaging in SAXS experiments results in a loss of structural information compared to high resolution crystallography. However, despite the lower resolution, SAXS has a number of significant advantages in the study of biological objects: (i)—crystallization is not required; (ii)—samples are measured in solution, i.e., under conditions close to physiological ones; (iii)—the study of conformational diversity in biomacromolecules is possible; (iv)—the analysis of biological complexes (e.g., protein–protein, protein-DNA, etc.) is possible both per se and in response to changes in external conditions, and others. Thus, the application of SAXS is often the only way to obtain direct structural information about systems with a disordered distribution of density inhomogeneities. An important feature of small-angle scattering is also the ability of the method to combine SAXS data with those obtained by techniques such as atomic force microscopy (AFM), TEM, cryo-EM, NMR and others to build structural models, and this approach significantly increases and complements the level of structural information of SAXS-based models. In addition, it should be taken into account that when determining the structure using small-angle scattering, the inverse problem is solved, that is, the three-dimensional structure of the scattering object is reconstructed from the one-dimensional scattering curve. The solution to such a problem is ambiguous and therefore, to narrow the corridor of possible solutions, independent information obtained by other structural methods is necessary. One of the most appropriate complementary to SAS methods is atomic force microscopy (AFM).

Atomic force microscopy is a technique invented in the 1980s for material science and only more recently has the application extended towards life sciences. Initially it has been used to study samples in air or vacuum, with significant results. As the instrument has evolved, its measurement capabilities have expanded, making it possible to use AFM to study samples in liquid. The undoubted advantage of AFM is that it is based on simpler physical principles than X-ray crystallography. In addition, AFM instruments are mechanically and electronically quite simple compared to electron microscopes [36]. Thus, visualization of individual and potentially more disordered assemblies can provide unique information about the structural biology of viruses. Moreover, AFM allows for characterization of the mechanical properties of the sample. It was shown that mechanical strength of the viral capsids may be related to the genome encapsulation mechanisms [37]. Finally, AFM can be used for direct investigation of dynamical processes, which help to understand the mechanisms of virus aggregation, viral adsorption to the host cells, etc. This information should be considered as an important part of the virus characterization.

In this review, we discuss recent advances in structural plant virology supported by SAXS and AFM, highlighting their advantages in resolving dynamic structural changes in solution for flexible filamentous viruses and intrinsically disordered viral proteins.

## 2. Structural Investigation of Helical Plant Viruses by Small-Angle X-ray Scattering

The use of SAXS in structural virology, particularly in the study of plant pathogens, is very promising. However, to the best of our knowledge, there is virtually no work in the scientific literature devoted to the study of helical plant viruses by small-angle scattering, and these samples have traditionally been studied by other techniques.

Despite the importance of the above achievements in the study of the structure of helical plant viruses, the study of their architecture, flexibility and self-assembly in water solution using the SAXS method is also certainly relevant. Here, we describe modern approaches developed in the field of small-angle scattering for the structural study of complicated biological objects, in this case plant viruses, and demonstrate the results of the studies.

### 2.1. Methods of SAXS Measurements, Data Analysis and Interpretation in Relation to Structural Study of Helical Plant Viruses

The measured scattering pattern in SAXS is basically a smooth, typically isotropic function *I*(*s*), where *s* = 4*π sinθ*/*λ* is the modulus of the scattering vector, or momentum transfer, and 2*θ* is the scattering angle. It should be emphasized that the measurement of the SAXS intensity *I*(*s*) is a complex technical problem due to the need to detect a relatively weak scattering signal in the small-angle range against the background of a high-power primary X-ray beam. Therefore, the full potential of SAXS has only been realized after the development of special small-angle cameras, two-dimensional detectors and the use of bright synchrotron radiation sources. Another important factor is the choice of methods for processing and interpreting SAS data. In a small-angle experiment, primary processing of the obtained data is very important. SAS uses the concept of a matrix, that is, the concept of a medium in which scattering object (sample) is located and which differs in its electron density from the electron density of the sample. To obtain scattering only from the sample, it is necessary to subtract from the total scattering the scattering from the matrix (solution, gel, solid, etc.). Therefore, a small-angle experiment consists of measuring scattering curves from a sample in a matrix and a matrix without a sample. All scattering curves are measured multiple times, then averaged, and the matrix is subtracted. This is a classic design for a small-angle experiment and primary data processing.

Currently, there are many software systems in the world designed for interpretation of SAS data. The most used and well-known of them are presented in Table 1 below with links to Internet resources.

The ATSAS software package is currently one of the most successful and widely used in the world [38]. Initial data processing, including the averaging of specified number of scattering curves, subtraction of the buffer signal, and calculation of structural SAXS invariants requires PRIMUS program, included in ATSAS suit [47].

Based on isotropic scattering curves, very important integral properties of the scattering particles, called invariants, can be determined directly from the scattering curves without using any a priori information. The invariants include geometric and weight parameters such as the radius of gyration *R_g_*, the intensity of scattering at zero angle *I*(0), the maximum size of scattering objects *D_max_*, the Porod volume (excluded volume) *V_p_*, molecular weights *MW* and the distance distribution function *p*(*r*).

The values of the forward scattering *I*(0), radii of gyration *R_g_* and the maximum particle diameter of the *D_max_* can be calculated from the distance distribution functions *p*(*r*) related to the scattering intensity *I*(*s*) by Fourier transform. The *p*(*r*) function can be evaluated by the GNOM program [48] as:

(1) $$p\left(r\right)=\frac{1}{2\pi^{2}}\int_{0}^{\infty }srI(s)\sin(sr)ds$$

In a homogeneous particle, the function *p*(*r*) is the histogram of the number of segments between any two points in the range between *r* and *r + dr* on the interval [0, *D_max_*], where *D_max_* is the maximum distance between two points within the scattering particle. Therefore, the calculated distribution of all pair distances within a scattering object contains information about the shape and structure of the particle and makes it possible to estimate its maximum size *D_max_* based on the condition *p*(*r*) *=* 0 at *r* > *D_max_*.

The molecular weights *MW* of the protein components of the multiphase models can be determined from the corresponding phase volumes *V_p_*, using an empirical coefficient of 1.65 (*MW* = *V_p_*/1.65) proposed in the work [49].

Given the fundamental ambiguity of solving small angle scattering inverse problems, the definition of invariants significantly reduces the range of possible solutions. Therefore, their determination is a primary task in the analysis of SAXS data. However, the ambiguity of the shape reconstruction for a given scattering profile can be preliminarily estimated using the AMBIMETER program. [50]. This is necessary for a qualitative assessment of the structural analysis performed.

The function *p*(*r*) is used for the ab initio shape reconstruction of monodisperse scattering particles in the homogeneous approximation by the bead modelling method [51]. In this method, the low-resolution shape is reconstructed by minimizing the target function by simulated annealing in the search volume of diameter *D_max_*, resulting in a compact interconnected ensemble of dummy atoms (beads). The scattering from these beads should match the experimental data. This approach has been implemented in the DAMMIN program [51]. It uses simulated annealing to build models that fit the experimental data *I_exp_*(*s*) to minimize the discrepancy:

(2) $$\chi^{2}=\frac{1}{N-1}\sum_{j}{\left[\frac{I_{exp}\left(s_{j}\right)-{cI}_{calc}\left(s_{j}\right)}{\sigma \left(s_{j}\right)}\right]}^{2},$$

where *N* is the number of experimental points, *c* is a scaling factor and *I_calc_*(*s_j_*) and *σ*(*s_j_*) are the calculated intensity from the model and the experimental error at the momentum transfer *s_j_*, respectively.

If the scattering object is inhomogeneous and contains parts with different electron densities, a more universal multiphase ab initio approach to the structure reconstruction is used. In the case of plant viruses consisting of proteins and RNAs, the two-component (two-phase) model can be built using the MONSA program [51]. In this multiphase ab initio modelling the complex particle is represented as an ensemble of several types of beads with different contrasts on a dense hexagonal grid within the search volume prescribed by the user. In the case of plant viruses, there are two types of non-zero contrast beads: one corresponds to the CP part, the other ones to the viral RNA. In such a multiphase approach, not only the overall shape of the whole particle is determined but also the details of its internal structure are highlighted: protein part is separated from the nucleic acid.

The appearance of Bragg peaks on the scattering curve indicates the internal ordering of the sample, i.e., the formation of quasi-crystalline regions with a repeating fragment of the structure. To analyze such kind of the SAXS curves the PEAK program [47] can be used. The program allows to determine the size of the crystalline region *L* using value (in radians) of *β_s_* which is the width at the half maximum intensity of the first Bragg peak at the angle 2*θ*_1_:

(3) $$L=\frac{\lambda }{\beta_{s}\cos\theta_{1}}$$

The degree of the sample disordering Δ/*d*_1_ is:

(4) $$\Delta /d_{1}=\frac{1}{\pi }\sqrt{\frac{\beta_{s}d_{1}}{\lambda }},$$

where *d*_1_ = 2*π*/*s*_1_ is the period of the ordered structure or interplane distance; and Δ is the standard deviation between the nearby regularly packed motifs.

Rigid body modelling of biological complexes can be performed by the MASSHA program [52] using known high-resolution structures of fragments of the complex. The CRYSOL program [53] was applied to calculate the theoretical scattering intensity from the reconstructed models.

### 2.2. Structural Study of Helical Plant Viruses Based on SAXS Measurements

As for structural studies of helical plant viruses using SAXS, there are very few works in the literature and these works relate only to the study of rod-shaped viruses. Some of them are listed below. The TMV virions are examined by SAXS on the small-angle beamline BM29 (ESRF, Grenoble) by Costa et al. [54]. The main focus of this work is to develop a protocol for the combined use of AFM and SAXS to determine possible sample radiation damage. The temperature dependence of the structure of TMV CP aggregates is investigated by Hiragi et al. on the small-angle beamline (Photon Factory, Japan) [55]. The kinetics of reassembly of cucumber green mottle mosaic and TMV virions is investigated by Sano et al. [56]. They show that the rate of TMV assembly is significantly higher. The kinetics of repolymerization of TMV CP in acidic medium is also investigated by Potschka et al. on the DORIS-I storage ring (DESY, Hamburg) [57]. Taken together with other kinetic information obtained in this work, the data suggest that polymerization of TMV CP under viral self-assembly conditions may proceed via a single-layered helical core containing about 20 subunits.

**Figure 1:**
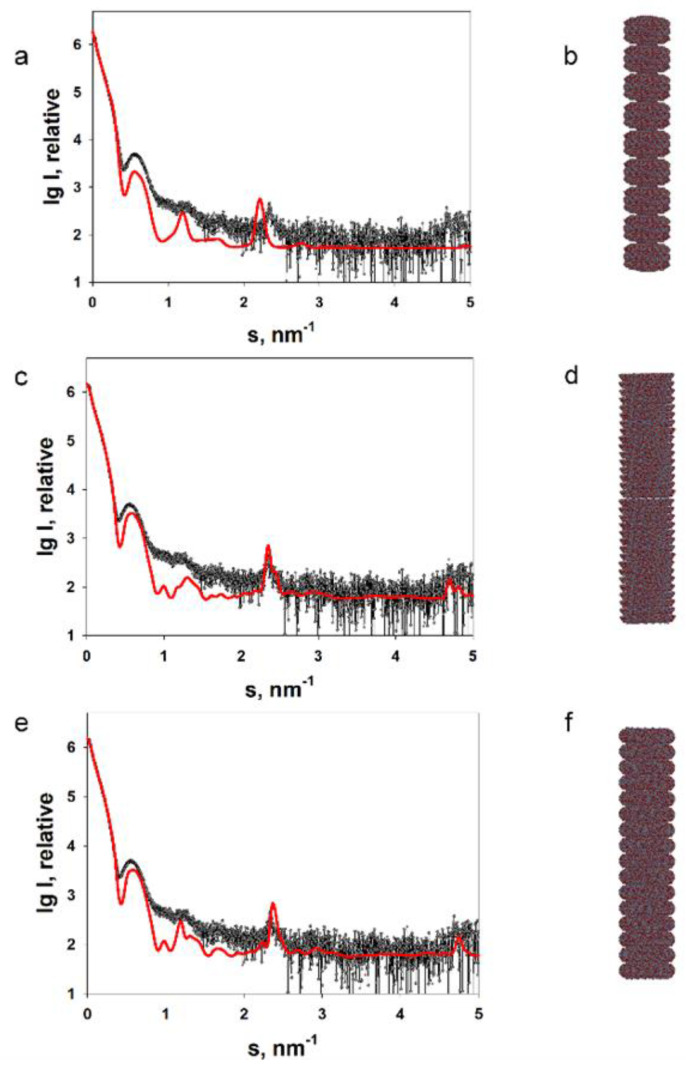
Experimentally obtained SAXS profile of repolymerized TMV CP stacked discs (**a**,**c**,**e**) and calculated SAXS data for the corresponding model structures (**b**,**d**,**f**). The scattering was calculated using the CRYSOL program. The structures of the replicated crystallographic model (**b**), the lower pair of discs (**d**) and the central pair of discs (**f**) were modelled with the MASSHA program. Points, experimental data; solid lines, calculated scattering of the model structures. Reprinted from [58], Copyright (2020), with permission from Springer Nature.

Below we present some of the most interesting, in our opinion, results we have obtained during structural studies of a number of plant viruses using small angle X-ray scattering. These synchrotron SAXS measurements have been performed at the European Molecular Biology Laboratory (EMBL) at the PETRA III storage ring (DESY, Hamburg) on the EMBL-P12 beamline equipped with a robotic sample changer and a 2D photon counting pixel X-ray detector PILATUS-2M (DECTRIS, Switzerland) [38]. We were able to analyze the structure of TMV virions in detail at a concentration of 1–4 mg/mL in solution, which is much lower than the concentration used in the study by Costa et al. (26 mg/mL) [54]. The CPs of TMV assemble into VLPs—stacked disk aggregates—at neutral pH. We have analyzed their structure in solution [58]. The TMV CP stacked disks are intriguing objects because of their amyloid-like stability. We have constructed a number of TMV CP stacked disc models from the upper/lower and central rings (from a high-resolution crystallographic model) and compared the scattering patterns calculated from them with the experimental ones. The best agreement with the experimental data was obtained for a model composed of the central ring pair of crystal model PDB:1EI7 (Figure 1). The radius of gyration of the TMV CP stacked disc model was close to the radius of the TMV CP aggregates calculated by Potschka et al. [57].

**Figure 2:**
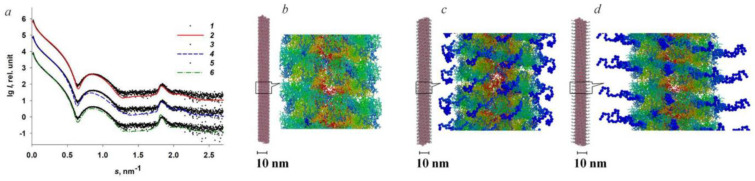
Approximation of the SAXS curves for the PVX virion. (**a**) Scattering from the PVX virion (curves *1*, *3* and *5*; experimental data); scattering from the original helix model (curve *2*); approximation by the PVX model with compact ΔN peptide (curve *4*); approximation by the PVX model with extended ΔN peptide (curve *6*). (**b**) Original virion model; (**c**) model of the PVX helix with compact ΔN peptide; (**d**) model of the PVX helix with extended ΔN peptide. Amino acid residues: 1–32, blue; 33–64, light blue; 65–158, green; 159–190, yellow; 191–222, orange; 223–237, red. Inset: magnification, ×10. Reprinted from [59], copyright (2023), with permission from Springer Nature.

Recently, the low-resolution structure of the filamentous plant virions potex- and potyviruses has been obtained by SAXS under conditions close to the physiological ones. In the work [59], we studied the effect of the flexible N-terminal domains CP on the structure and physicochemical properties of the flexible virions of the potexviruses PVX and alternanthera mosaic virus (AltMV) in solution by SAXS. These proteins differ in the presence of the N-terminal domain of 28 residues (∆N peptide) in the PVX CP. It was found that the CP PVX are more ordered; in particular, the PVX CP has a greater proportion of crystalline regions. We used the SAXS data and available high-resolution structures of the virion fragments to model the whole viral particles in solution. The ΔN peptide was added to the atomic model of PVX CP. The diameters of the PVX and AltMV virions and the helix parameters were calculated from the SAXS data. The influence of the structure of the ΔN peptide and its position on the virion structure was analyzed (Figure 2).

The small deviations of the model curves from the experimental SAXS data, which remained even after the addition of the ΔN peptide, showed that the structure of the flexible regions in individual PVX CP subunits can vary, i.e., virions with compact and extended ΔN peptides can coexist in solution. In the compact conformation models, the ΔN peptides are partially adjacent to the virion surface, suggesting their interaction with the adjacent subunits. The melting point of PVX virions was 10–12 °C higher than that of AltMV virions. Presumably, the increased thermal stability of PVX virions compared to AltMV is provided by the extended ΔN peptide, which forms additional interactions between the CP subunits in the PVX virion.

**Figure 3:**
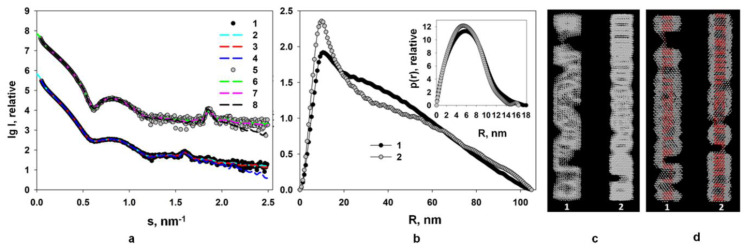
(**a**) Curves *1* and *5* are the experimental ones; curves *2* and *6* were calculated with the GNOM program based on the distance distribution functions *p*(*r*); curves *3* and *7* are the scattering profiles calculated from the ab initio models reconstructed with the DAMMIN program; curves *4* and *8* are scattering profiles calculated from the multiphase ab initio models reconstructed with the MONSA program for PVA and PVAΔ32, respectively. For better visualization the scattering curves from PVA and PVAΔ32 are shifted vertically relative to each other; (**b**) calculated with GNOM program distance distribution functions from PVA (*1*) and PVAΔ32 (*2*). Inset: distance distribution functions *p*(*r*) in the cross-sectional analysis mode calculated for PVA (*1*) and PVAΔ32 (*2*); (**c**) shapes of PVA (*1*) and PVAΔ32 (*2*) virions reconstructed with the DAMMIN program; (**d**) shapes of PVA (*1*) and PVAΔ32 (*2*) virions reconstructed with the MONSA program. Protein coat is presented as grey virtual atoms (beads), viral RNA—as red beads. Reprinted from [60], copyright (2021), with permission from Springer Nature.

Potato virus A (PVA) belongs to the family Potyviridae. Similar to PVX described above, it also contains a partially disordered N-terminal domain within its CP. We have investigated the structure of intact PVA and partially trypsinized (PVA∆32) virions using small-angle X-ray scattering and complementary techniques [60]. It is shown that after removal of 32 N-terminal residues of the CP, the potyvirus virion saved compact shape, while the helical pitch of the CP packing is changed. Based on the SAXS data, we performed ab initio modelling, including a multiphase procedure, and reconstruction of the PVA structure using available high-resolution structures of the homologous CP PVY. A low-resolution structure of the filamentous intact and PVA∆32 virus was identified for the first time (Figure 3).

According to the TEM data, the PVA∆32 virions are rod-shaped, in contrast to the flexible particles of the intact virus [60], indicating an increase in the compactness and stability of the PVA∆32 particles. The TEM data correlate well with the SAXS data as well as the new tritium planigraphy results [61].

Thus, our structural analysis indicates the major role of the N-terminal domain of the CP of potato viruses A and X in maintaining the necessary flexibility of the virions. It is noteworthy that the SAXS data obtained are in good agreement with those previously obtained by other authors using cryo-EM [27]. According to the calculations of the accessible surface areas of the CP inter-subunit contacts carried out on the basis of the cryo-EM model of the BaMV potexvirus [27], it can be concluded that the inter-subunit contacts are represented in almost equal proportions by the protein core domains and the N-/C-terminal extensions. At the same time, the inter-subunit CP contacts within the flexible BaMV virion occupy almost the same surface area as the inter-subunit CP contacts within the rod-shaped TMV virion, confirming the role of the N-/C-terminal extensions in ensuring virion flexibility [27,61].

We have also used SAXS to characterize and study the dissociation of VLPs without RNA of the PVA CP [62,63]. We have shown that in a pH 7.8 buffer, the potyvirus PVA CP sample represents a mixture of short-order VLPs, consisting of approximately 60 subunits. However, in the pH 10.5 buffer containing 0.5 M NaCl, the protein sample contained oval particles of 20–30 nm diameter consisting of 30 subunits. These particles are readily reassembled into regular VLPs by changing the pH back to neutral. It is possible that these particles represent some kind of intermediate in PVA assembly in vitro and in vivo. Analysis of the kinetics of VLP assembly has shown that electrostatic interactions play a role in this process [62,63].

In summary, low resolution spatial features of a number of plant viruses in solution (i.e., under near physiological conditions) have been obtained using small angle X-ray scattering. The SAXS profiles have revealed a number of structural features of the viral particles: their shape, diameter, helix pitch and periodicity of the structures of virions and VLPs. Particular attention has been paid to the structural study of the N-terminal regions of the coat proteins of potato viruses A and X. These regions have been shown to play an important role in the formation of the helical structure of the virions and their flexibility. As previously discovered, the deletion or modification of the N-terminal peptide composition affected its activity as a translational repressor [64]. Understanding the structural features of flexible plant viruses and the role of N-terminal domains in their coat proteins is necessary for the construction and further application of virus particles and virus-like nanoparticles as a platform for epitope presentation and vaccine development [4].

## 3. Atomic Force Microscopy Investigation of Plant Viruses

A prerequisite for the study of viruses by AFM is their immobilization on the surface of the substrate, which should be sufficiently smooth compared to the size of these viruses. The most commonly used substrates for the AFM of viruses are mica and highly-oriented pyrolytic graphite (HOPG), which are characterized by the presence of extended regions of atomic smoothness [65], as well as glass, silicon, and other surfaces [66,67].

AFM has many modifications and modes of operation, depending on the type of “probe-sample” interaction and the detected parameter underlying the feedback operation. The contact mode of scanning mentioned above is rarely used to study individual virions due to the relatively high interaction forces of the cantilever with the sample, both in the vertical and horizontal directions, which can lead to mechanical destruction of a virion or its “sweeping away” from the substrate surface. Nevertheless, the contact mode can be used to study virus-based crystals, because the viral crystal is much stronger than individual virions and each virion is well fixed in the crystal [65]. For example, the contact mode was used to study the AFM growth of a two-dimensional crystal of satellite tobacco mosaic virus (STMV) [68].

**Figure 4:**
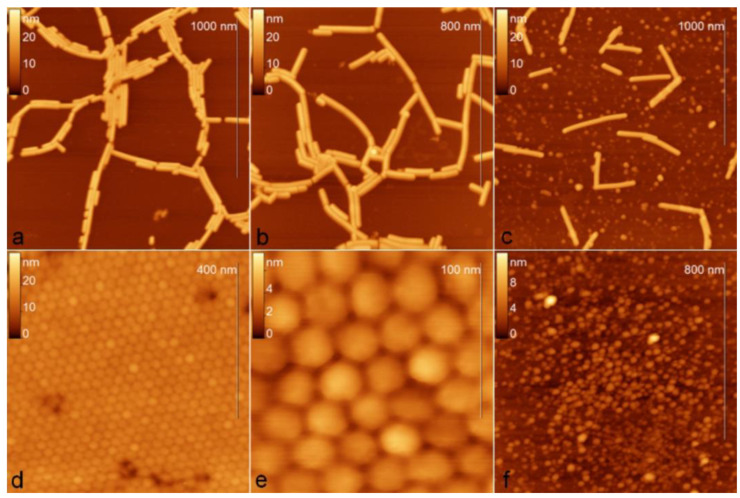
Tapping mode AFM images of (**a**) *Poa* semilatent virus, (**b**) barley stripe mosaic virus, (**c**) tobacco mosaic virus, (**d**,**e**) brome mosaic virus, and (**f**) alfalfa mosaic virus. Reprinted from [71], copyright (2007), with permission from Elsevier.

Perhaps the most commonly used AFM mode for studying the structure of viral particles is the intermittent contact (tapping) mode [69]. In this mode, the cantilever is constantly oscillated by the piezoelectric element at a frequency close to the resonant frequency. In this case, the feedback is maintained by the amplitude of the cantilever oscillations, which changes as the probe interacts with the surface. The main advantage of the intermittent contact mode over the contact mode is the lower interaction force between the probe and the sample (both in the normal and lateral directions), which allows more stable AFM imaging of individual virus particles. In addition, the intermittent contact mode produces an additional phase image (the difference in the phase of the oscillations of the driving force and the cantilever), which often has a higher resolution than the topography or amplitude [70]. The tapping mode AFM images of five plant viruses are shown in Figure 4 [71]. Among them are three filamentous viruses, *Poa* semilatent virus (PSLV), barley stripe mosaic virus (BSMV), and TMV possessing helical symmetry (Figure 4a–c), and two other viruses from family Bromoviridae, which demonstrate another type of morphology: an icosahedral brome mosaic virus (BMV) from genus *Bromovirus* (Figure 4d) and a bacilliform alfalfa mosaic virus from genus *Alfamovirus* (Figure 4e).

**Figure 5:**
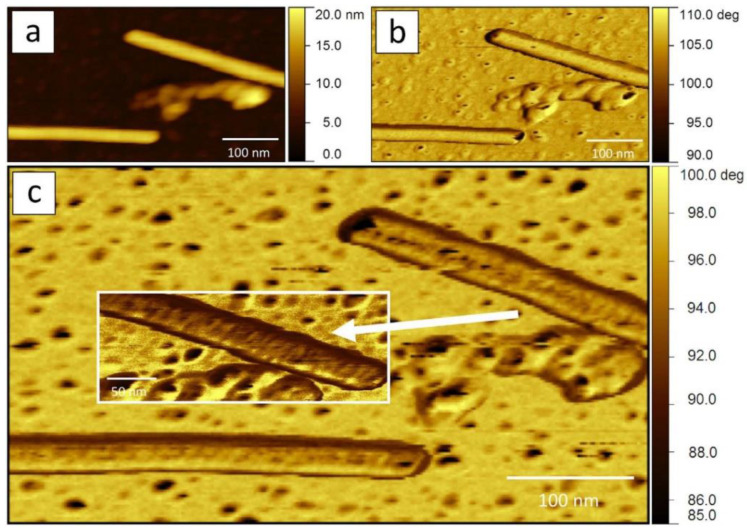
Standard and multi-frequency AFM images of TMV virions: (**a**) height image; (**b**) phase shift image of the first excited mode (115 kHz); (**c**) phase shift image of the second excited mode (720 kHz). Reproduced from [72] under the CC BY license (https://creativecommons.org/licenses/by/4.0/ (accessed on 7 March 2024)).

A variation in the intermittent contact mode is multi-frequency AFM, in which the cantilever is oscillated at several frequencies simultaneously and the amplitude and phase images at these frequencies are analyzed separately. AFM images acquired at higher frequencies and lower amplitudes tend to better distinguish surface regions with different properties, thus providing more morphological detail on the surface of the virion (Figure 5) [72,73].

Another group of AFM modes whose use in virus research is growing are those based on the force curve, i.e., the dependence of the cantilever deflection or, up to a multiplier, the force of the cantilever interaction with the sample, on the z-coordinate of the sample. Such modes (e.g., PeakForce, Quantitative Imaging, Hybrid Mode, etc.) bring the probe to the sample at each point of the surface grid with the maximum force (cantilever deflection) set by the operator, and the topography of the sample surface is formed from the z-coordinate of the sample. The force curve-based AFM modes allow the interaction force between the cantilever and the sample to be further minimized (down to ~50 pN [74]), thereby increasing the resolution of images on soft objects.

**Figure 6:**
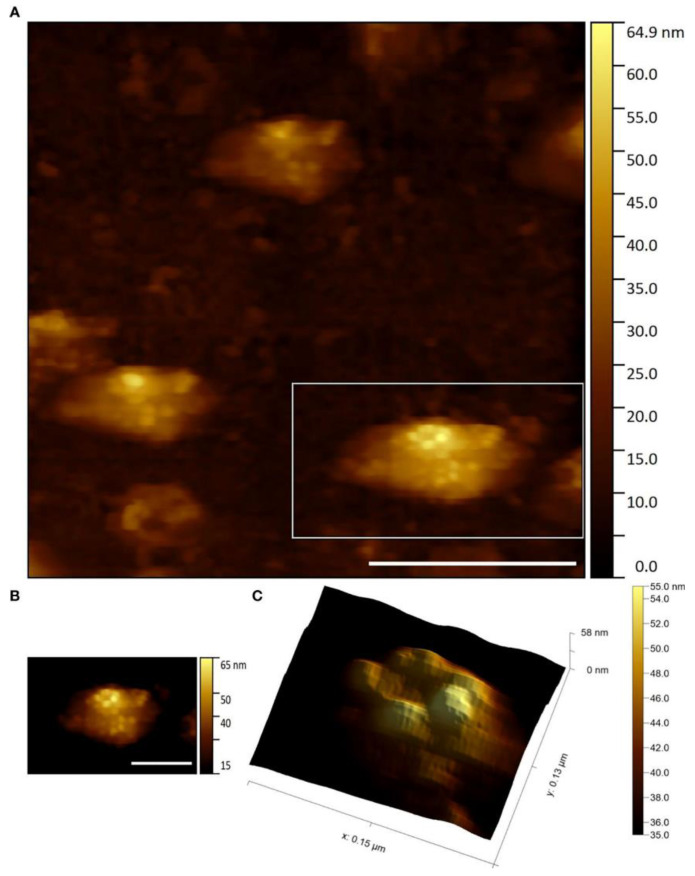
Capsid protein structure of EhV-86 particles visualized by AFM: (**A**) height image (scale bar 400 nm); (**B**) height image (scale bar 200 nm); (**C**) 3D rendering of the height image with illumination; (**B**,**C**) represent the field marked in (**A**) by a rectangle. Reproduced from [81] under the CC BY license (https://creativecommons.org/licenses/by/4.0/ (accessed on 7 March 2024)).

AFM studies can be carried out in both air and in aqueous solutions, which is an additional advantage for the study of viruses. AFM resolution on soft objects, such as proteins and viruses, can be up to ~1 angstrom in the vertical direction and ~5 angstroms in the lateral direction both in air and in liquid [75]. When applied to viruses, this means that AFM can not only visualize individual virions, but also distinguish their shape and the structural features of capsid proteins. Using AFM, several authors revealed a thin structure of various viruses belonging to different plant virus families possessing different types of symmetry. For example, the AFM technique has identified the “beads-on-a-string” structure of PVX [76] and PVA [77], characterized the helical folding of the TMV coat [72,78], and has revealed the patterns of capsomeres of *Paramecium bursaria* chlorella virus type 1 (PBCV-1) [79] from genus *Chlorovirus*, family Phycodnaviridae, a rather rare group of plant viruses with double-enveloped virions having icosahedral protein capsids positioned between the two lipid membranes, which infect algae; icosahedral turnip yellow mosaic virus (TYMV) from genus *Tymovirus*, family Tymoviridae; spherical cauliflower mosaic virus (CaMV) from genus *Caulimovirus*, family Caulimoviridae; icosahedral bromovirus (BMV) [78,80] and a double enveloped *Emiliania huxleyi* virus 86 (EhV-86) [81] (Figure 6) from genus *Coccolithovirus*, family Phycodnaviridae. The AFM method has been used to characterize the artificial ribonucleoproteins (RNPs) assembled in vitro from PVX CPs and foreign viral RNAs (originated from TMV, AltMV, or other viruses). Some RNPs had “heads” and “tails” consisting of free RNA molecules that looked like elongated strands (so-called single tail particles). Using the AFM method, it is possible to reliably quantify the length and diameter of such RNPs and compare their morphology with that of homologous RNPs and native virions [82].

Maximum spatial resolution is usually achieved on virus crystals [78,80]. AFM of the surface of a viral crystal makes it possible to determine the lattice parameters with high accuracy. AFM of the surface of a virus crystal allows the lattice parameters to be determined with high accuracy. For example, AFM has allowed the parameters of the cubic and orthorhombic lattices of the smallest spherical virus, STMV, to be determined. The distance between the centers of the virus particles was 18 and 17 nm, respectively, which correlates well with X-ray diffraction data [78]. The periodicity of a two-dimensional BMV crystal according to AFM data was ~28 nm [71,78], which also agrees with the diameter of this virion known from crystallography (28 nm).

The major advantage of AFM over other high resolution microscopy methods is the ability to study dynamic processes in a liquid. For example, the growth of STMV crystals on the surface of silicon has been visualized using AFM in solution [66,68], and the binding process of lytic double-stranded DNA-containing EhV with the host cell, the algae *Emiliania huxleyi*, has been visualized in an aqueous medium using high-speed AFM [81].

The ability to study the mechanical properties of virus particles is another important feature of AFM. Various mechanical properties of the virus can be obtained from so-called nanoindentation experiments, i.e., controlled compression of a virus particle with a cantilever to a given maximum force, followed by its withdrawal from the surface. Nanoindentation experiments are accompanied by the recording of force curves, from the analysis of which it is possible to determine the type of deformation (elastic, plastic), the strength of the virus particle (the maximum force required to destroy the particle), and also to calculate a number of parameters such as stiffness, deformation, adhesion, modulus of elasticity, etc. Nanoindentation experiments can be performed using the force curve-based operating modes mentioned above, where the mechanical parameters are obtained simultaneously with the sample topography (mechanical mapping) [74]. It should be noted that in order to obtain some specific mechanical properties of a virus particle from force curves, such as spring constant or modulus of elasticity, it may be necessary to select a physical model and to know the additional parameters, such as probe geometry, Poisson’s ratio, etc. [83].

The CCMV nanoindentation experiments revealed the presence of a reversible linear region of the dependence of the cantilever load on the z-coordinate of the specimen, corresponding to the elastic mode of viral deformation [67]. This region was maintained up to a deformation of 20–30% of the viral diameter and an applied force of 0.6–1 nN. As the cantilever pressure on the virus particle increased, a sharp decrease in the cantilever pressure force was observed, associated with irreversible plastic deformation of the particle. It was shown that the average values of spring constant and threshold force of elastic deformation for the wild-type CCMV virion (0.2 N/m and 0.81 nN, respectively) were higher than those for the empty CCMV capsid (0.15 N/m and 0.6 nN, respectively). It was also shown that a point mutation in the CCMV envelope protein increased the strength of both the viral particle and the empty capsid. The data obtained allowed the conclusion that the strength of the viral capsid is determined not only by its structure, but also by its interaction with the genome [67]. Using nanoindentation experiments, Wilts et al. have demonstrated swelling and a significant decrease in rigidity of the CCMV virion with an increase in pH from 4.8 to 7.5, which may be due to the peculiarities of the release of the genetic material of this virus [84]. Hernando-Pérez et al. have shown that even with a small compressive force on the BMV virion, plastic deformations can be observed over time, which are probably caused by the dynamics of defects in the viral capsid lattice [85].

Finite element modelling (FEM) and the Hertz model are used to determine the Young’s modulus of the virus particles from the force curves. The Young’s modulus of the empty CCMV capsid is 140 MPa (FEM) [67], while the radial Young’s modulus of the TMV virion is 0.92 ± 0.15 GPa (FEM) and 1.0 ± 0.2 GPa (Hertz model) [86]. In another work, a similar value of the radial Young’s modulus is obtained for the TMV virion coated with polyaniline, 1.266 ± 2 GPa [69].

In conclusion, a major advantage of the AFM method is not only the ability to work with samples in liquid, but also to study the dynamics and mechanical properties of individual viral particles in real time. In addition, the ongoing development of a variety of AFM modes, as well as the combination of AFM with other research methods (optical, fluorescence, etc.), is creating new and exciting opportunities for the structural study of viruses every year. Developments already underway are extending the ability to study individual viral particles and other protein cells in addition to classical EM and cryo-EM, X-ray crystallography and other structural methods.

## 4. Conclusions

The combination of biochemical, physicochemical and physical approaches has led to the elucidation of the high-resolution structure of virions and their components and has increased our knowledge of the viral life cycle, its interaction with the host cell and possible strategies to combat viral infections. This interdisciplinary interest in the study of viruses has attracted several powerful structural research methods down to the level of conformational rearrangements and dynamics of viral particles. However, the dynamic rearrangements of viral coat proteins and their interactions with cellular structures require study under physiological conditions using methods capable of visualizing the kinetics of the processes. These structural methods are atomic force microscopy and small-angle X-ray scattering, which have already been well established in structural studies of viruses, especially in combination [87,88,89]. To date, most of these studies have been carried out on plant viruses, which have dense capsids that allow the best spatial resolution even in solution.

Although the resolution of SAXS and AFM is lower than that of X-ray crystallography and cryo-EM, these methods are extremely useful in the study of flexible filamentous viruses, intrinsically disordered proteins that are often part of their capsids, and dynamic changes in viral structure upon environmental changes such as changes in pH, salts, osmotic pressure, etc. However, these methods are still underestimated in their ability to provide exceptional information on the dynamic behavior of viruses and their proteins in solution at the molecular level. Not long ago, SAXS was used for the first time to obtain a large amount of data on the structure of some helical plant viruses under physiological conditions. It has also been possible to relate the structure of individual parts of the capsid proteins to the properties of the whole virus particle, for example, in the case of the N-terminal regions of the envelope proteins of potato viruses A and X. In addition, a complementary AFM approach makes it possible to obtain mechanical properties of individual virus particles under physiological conditions and their changes in real time. Furthermore, the combination of AFM with optical and fluorescence microscopy allows the study of virus-cell interactions at the level of individual virions. All these advantages can be used to gain new insights into the viral life cycle and to use viruses as nanocontainers for drug delivery and vaccines.

It is important to note that, in addition to their structural function, coat proteins have many other important activities in the infection cycle of plant viruses and in the defense response of host plants to viral infection. The presence of intrinsically disordered regions within the coat proteins largely determines their multifunctionality. They are involved in the transport of the virus within the plant, in the determination of the host plant range and of infectivity, pathogenicity, and in the transmission of a virus from plant to plant by natural vectors. Coat proteins are also involved in the regulation of replication, transcription and translation of viral RNA [90]. New data on the structure of coat proteins, virions, and transport forms of helical plant viruses will allow understanding of the molecular mechanisms that provide multiple interactions between viruses and plants that deter-mine specific features of viral infection.

## Figures and Tables

**Table 1:** Software packages for biological SAS.

Program Package	Short Description
ATSAS [38] https://www.embl-hamburg.de/biosaxs/software.html (accessed on 7 March 2024)	Primary data processing; Ab initio modelling; Atomic structure based (molecular) modelling; Analysis of mixtures and flexible systems.
BioXTAS RAW [39] https://bioxtas-raw.readthedocs.io/en/latest/ (accessed on 7 March 2024)	Primary data processing; Utilization of ATSAS modules.
SASTBX [40] https://bio.tools/sastbx (accessed on 7 March 2024)	Building molecular shapes; Refinement of existing atomic models.
SASview [41] https://www.sasview.org (accessed on 7 March 2024)	Multipurpose data analysis package; Model fitting; Distance distribution function evaluation; Invariant analysis; Correlation Function Analysis.
ScÅtter https://bl1231.als.lbl.gov/scatter/ (accessed on 7 March 2024)	Primary data processing.
FoXS/FoXSDock/MultiFoXS [42] http://modbase.compbio.ucsf.edu/foxs (accessed on 7 March 2024)	Calculation of SAXS data from atomic coordinates, combined with docking or flexibility modelling.
SASfit [43] https://sasfit.org/ (accessed on 7 March 2024)	Primary analysis of SAS data; Integral structural parameters calculation; Size distribution evaluation.
US-SOMO [44] https://somo.aucsolutions.com/ (accessed on 7 March 2024)	Evaluation of hydrodynamic parameters from SAS-based models.
GENFIT [45] https://sites.google.com/site/genfitweb/ (accessed on 7 March 2024)	SAS data fitting by a set of more than 30 model types.
DENSS [46] https://tdgrant.com (accessed on 7 March 2024)	Ab initio modelling; Scattering calculation from atomic models.

## Data Availability

All the data are available on demand.
